# Tools and Strategies for Malaria Control and Elimination: What Do We Need to Achieve a Grand Convergence in Malaria?

**DOI:** 10.1371/journal.pbio.1002380

**Published:** 2016-03-02

**Authors:** Janet Hemingway, Rima Shretta, Timothy N. C. Wells, David Bell, Abdoulaye A. Djimdé, Nicole Achee, Gao Qi

**Affiliations:** 1 Liverpool School of Tropical Medicine, Liverpool, United Kingdom; 2 Malaria Elimination Initiative, Global Health Group, University of California, San Francisco, San Francisco, California, United States of America; 3 Medicines for Malaria Venture, Geneva, Switzerland; 4 Global Good Fund and Intellectual Ventures Laboratory, Bellevue, Washington, United States of America; 5 Department of Epidemiology of Parasitic Diseases, Malaria Research and Training Center, University of Bamako, Bamako, Mali; 6 Department of Biological Sciences, Eck Institute for Global Health, University of Notre Dame, Notre Dame, Indiana, United States of America; 7 Jiangsu Institute of Parasitic Diseases, Meiyuan, Wuxi, Jiangsu, People’s Republic of China

## Abstract

Progress made in malaria control during the past decade has prompted increasing global dialogue on malaria elimination and eradication. The product development pipeline for malaria has never been stronger, with promising new tools to detect, treat, and prevent malaria, including innovative diagnostics, medicines, vaccines, vector control products, and improved mechanisms for surveillance and response. There are at least 25 projects in the global malaria vaccine pipeline, as well as 47 medicines and 13 vector control products. In addition, there are several next-generation diagnostic tools and reference methods currently in development, with many expected to be introduced in the next decade. The development and adoption of these tools, bolstered by strategies that ensure rapid uptake in target populations, intensified mechanisms for information management, surveillance, and response, and continued financial and political commitment are all essential to achieving global eradication.

## Abstract 2

Malaria elimination in many settings will not be possible without new vaccines, drugs, and vector control products. Although a healthy pipeline of new products in each of these areas has been developed over the past decade, significant work is required to bring these products to market and deploy optimal strategies for their use.

## Introduction

The past decade has seen considerable progress in reducing preventable mortality in low- and middle-income countries (LMICs), as evidenced by the 50% reduction in childhood deaths, 25% reduction in malaria cases, and the World Health Organization (WHO) certification of four countries as malaria-free [1]. This progress has led to continued optimism and commitments by global and regional partners to commit to malaria elimination and eradication within a generation. WHO’s *Global Technical Strategy for Malaria* (*GTS*), recently endorsed by the World Health Assembly in 2015, and the Roll Back Malaria (RBM) Partnership’s *Action and Investment to defeat Malaria* (*AIM*) have embraced the goal of a “world free of malaria” and have put forward ambitious targets of reducing malaria case incidence and mortality rates globally by at least 90% by 2030.

However, there remain an estimated 3.2 billion people in 97 countries and territories at risk of malaria infection. About 214 million malaria cases were estimated to occur in 2015, leading to 438,000 deaths, and key challenges exist to sustaining and improving on recent gains [1]. Research-and-development spending on drugs, vaccines, and basic research more than quadrupled between 1993 and 2013, reaching US$550 million annually. However, continued investment in research and development and rapid deployment of new tools is needed (see Box 1). The plasticity of the mosquito and the *Plasmodium* parasite has led to increasing resistance to medicines and insecticides. Resistance to artemisinin-based combination therapies (ACTs) has been detected in five countries in Southeast Asia. The spread of these strains to Africa or the Indian subcontinent could be catastrophic. In Africa, resistance has been detected against two or more insecticides in two-thirds of countries where malaria is endemic. Up to 80% of infections are asymptomatic, and *Plasmodium vivax* parasites remain dormant for months or even years after initial infection. Current field tests are not sensitive enough to pick up the low density of parasites in low-transmission areas.

**Box 1. Five Key Papers in the Field**

1. Nikolaeva D, Draper SJ, Biswas S. Toward the development of effective transmission-blocking vaccines for malaria. Expert Rev Vaccines. 2015 Jan 19:1–28.
2. Price RN, Nosten F. Single-dose radical cure of *Plasmodium vivax*: a step closer. Lancet. 2014 Mar 22;383(9922):1020–1.
3. UNITAID. Malaria Diagnostics Landscape Update Technical Report. Geneva, Switzerland: World Health Organization, 2015 February 2015. http://www.unitaid.eu/images/themarketshare/Malaria_Diagnostics_Landscape_Update_Fe_2015.pdf
4. Wells TN, van Huijsduijnen RH, Van Voorhis WC. Malaria medicines: a glass half full? Nat Rev Drug Discov. 2015;14(6):424–42.
5. Hemingway, J. The role of vector control in stopping the transmission of malaria: threats and opportunities. Phil Trans. Roy Soc. B. 369 20130431 2014 10.1098/RSTB.2013.0431

As transmission decreases, it is increasingly clustered in at-risk populations such as forest workers, who often migrate among job sites, taking the disease with them; or geographically resistant areas or “hotspots” such as swamps and other sources of stagnant water that serve as breeding sites.

The *Lancet* Commission on Investing for Health determined that if the right investments are made in scaling up existing health interventions and in developing new prevention, treatment, and surveillance tools, the world could achieve a “grand convergence” by 2035, with preventable deaths reaching universally low levels and economic benefits exceeding cost by a factor of 9–20 [2]. Historically, LMICs that have aggressively adopted new tools have seen an additional 2%-per-year decline in child mortality rates compared with nonadopters [2]. However, adoption alone of new and existing tools with poor implementation will have little impact on disease transmission in the long term. The difficulty of maintaining major declines in disease following effective malaria control initiatives underscores the fragility of these successes [3]. Sustained success will require the combination of universal coverage of interventions with implementation of a robust surveillance system that collects, transmits, and analyzes data about cases and program activities in real time to inform rapid response strategies [4–6]. However, detection and response remain serious operational challenges in resource-constrained settings plagued by weak health and information systems and uncertain funding streams.

Effective and efficient scale-up of existing interventions is required, which includes the early treatment of malaria cases with ACTs, intermittent preventive treatment for pregnant women (IPTp), and interventions that reduce human–vector contact, such as indoor residual spraying (IRS) or use of long-lasting insecticide-treated bed nets (LLINs).

Gains will require not only new approaches for scaling up existing strategies for malaria treatment and prevention to address areas of high malaria transmission and interrupt parasite transmission, but also novel tools to counter the growing threat of drug and insecticide resistance and better surveillance mechanisms to more efficiently target interventions to populations and areas of high risk of malaria transmission.

We describe the tools and strategies that are currently being used, as well as those on the near-term horizon, that have the potential to accelerate the decrease in malaria mortality and, thus, bend the curve. We also identify opportunities and gaps in current research.

## Potential Game Changers on the Horizon

**Fig 1:**
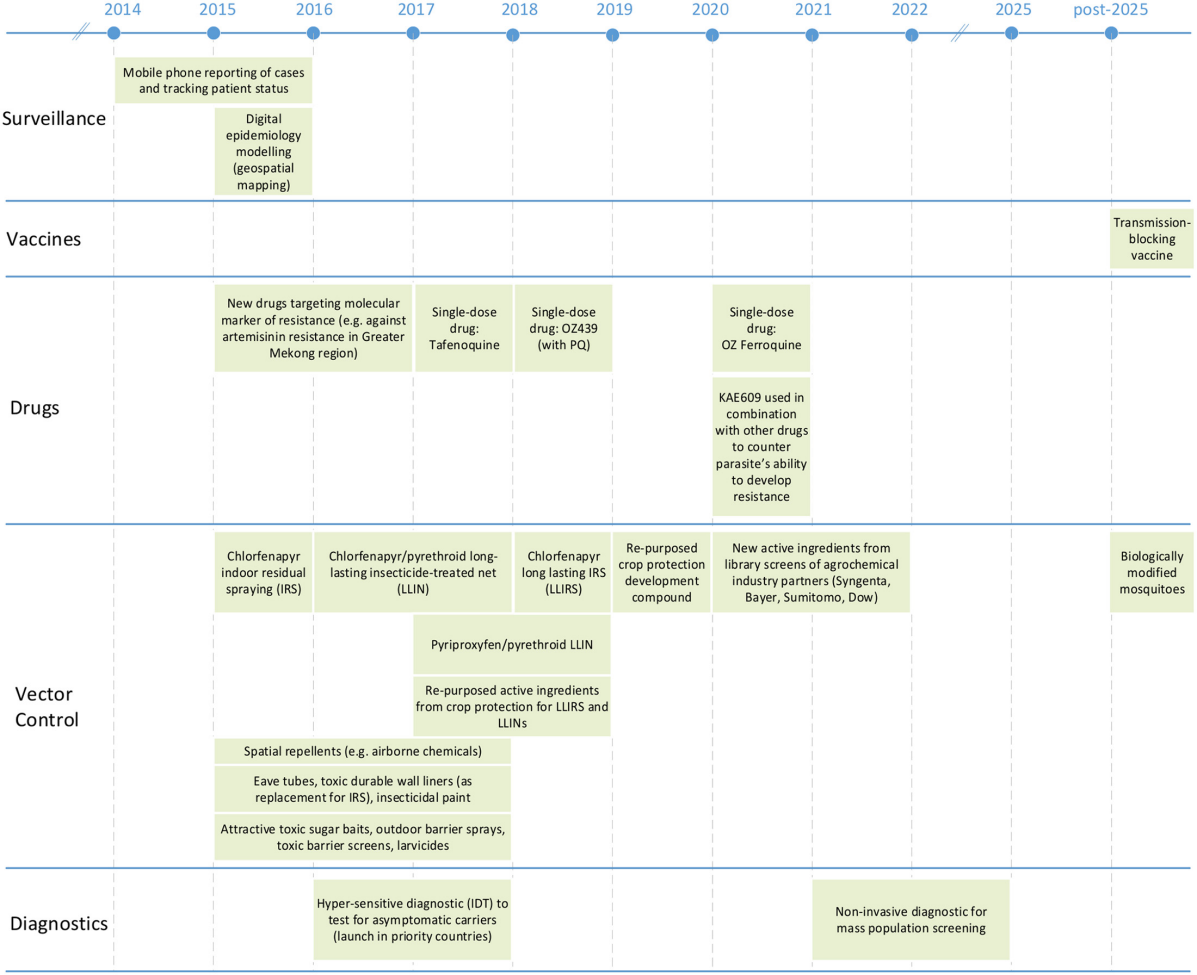
Projected time periods for introduction of novel malaria technology. Accelerating toward eradication through innovative tools that monitor (surveillance), prevent (vector control, vaccines), and treat (drugs, diagnostics) malaria.

The current malaria landscape offers both opportunities and threats. Amongst the opportunities are new products that provide a step change in our ability to diagnose, prevent, or treat malaria (Fig 1). There are many threats, but the most significant is that selective pressure will result in drug-resistant parasites or insecticide-resistant vectors that reduce the effectiveness of existing interventions. Malaria control and elimination demand new tools and technologies as well as better mechanisms for maintaining detailed surveillance and spatial decision support systems (SDSS) that improve reporting and timeliness of activities [7].

The majority of new products currently under development are in partnership with Product Development Partnerships (PDPs) such as the Program for Appropriate Technology in Health (PATH); Malaria Vaccine Initiative (MVI), Medicines for Malaria Venture (MMV), the Innovative Vector Control Consortium (IVCC), the Foundation for Innovative New Diagnostics (FIND), Novartis Institute for Tropical Diseases (NITD), Drugs for Neglected Diseases Initiatives (DNDi), and the European Vaccines Initiative (EVI).

There are over 100 products in the research and development pipeline that will benefit regional elimination and global eradication goals. These range from innovative diagnostics, medicines, vaccines, and vector control products to improved mechanisms for surveillance and targeted responses. Many of these promising products are expected to be introduced within the next ten years (Fig 1).

## Diagnostics

Improved diagnostics are needed to monitor and measure changes in infection rates, assure the quality of medicines and insecticides, and measure characteristics that drive treatment choice. Current-generation Rapid Diagnostic Tests (RDTs) are excellent for diagnosing people with symptomatic malaria, who have relatively high parasite densities. They have expanded access to malaria diagnosis outside health facilities to more peripheral communities beyond the reach of microscopy. However, a critical step in reducing the infectious burden of the disease is missing: current tests cannot detect either the low-level blood-stage infections of any malaria species or the dormant liver stages of *P*. *vivax* and *P*. *ovale* [8]. A highly sensitive point-of-care field test is needed to rapidly detect low-density parasitemia and identify all infected individuals, enabling immediate treatment.

In addition, improved point-of-care diagnostics for other causes of fever, particularly multiplexed with malaria tests, will be increasingly important to address the burden of other acute febrile disease and, in so doing, maintain the viability and legitimacy of the continued intensive screening for malaria necessary to drive down residual transmission.

### Point of Care G6PD Deficiency Tests

*P*. *vivax* can be cured by 8-amino quinolones, such as primaquine [8]. However, treating G6PD-deficient patients with primaquine is associated with a risk of acute hemolytic anaemia. As such, it is necessary to detect individuals deficient in the G6PD enzyme to facilitate safe and effective treatment in patients with *P*. *vivax*. These tests are currently available only in limited facilities [9]. The immediate availability of information on enzyme deficiency, through rapid testing and good access to data, will enable the scale-up of effective *P*. *vivax* treatments to drive malaria transmission down.

### Next Generation RDTs and Screening Tests

The Infection Detection Test (IDT) is aimed at a limit of detection an order of magnitude lower than that of current RDTs while maintaining affordability and ease of use. It has the potential to detect infections hidden from current case management tests and plays a major role in elimination settings. The development of field-deployable survey tools such as serology and parasite-detecting tests that can identify population hot spots will enable more effective targeting of intensive screening, mass drug administration, and other interventions aimed at residual foci of transmission.

### Reference Methods

As countries approach malaria elimination, the proportion of low-density and asymptomatic infections increases, and new reference standards that detect antigens, nucleic acids, or other biomarkers are needed for diagnosis and tracking changes in the rate of malaria infections. There are two main molecular methods available: Loop-mediated isothermal amplification (LAMP) and polymerase chain reaction (PCR). These are capable of detecting low-density parasitemia, while PCR may also distinguish between local and imported cases. While their field applicability remains limited, the cost and complexity of these assays are decreasing.

### Insecticide Quantification Kits

Simple point-of-use kits have been developed to allow quality assurance of insecticide formulations and field-based measurement of IRS quality. These need to replace insecticide bioassay methods, which are impractical in resource-limited public health systems and rarely used in operational programs despite being recommended as the gold standard.

## Medicines for the Treatment and Prevention of Malaria

The overall goal of new medicines is twofold. First, it is important to have new medicines available that are active against emerging resistant strains of the parasite. Second, as part of the malaria elimination agenda, simpler courses of treatment would be useful. Over the last decade, five new fixed-dose ACTs have been launched, of which only one (artemether-lumefantrine) is available as a child-friendly taste-masked formulation. Over the next few years, more pediatric ACT versions will be launched. The availability of five different partner drugs is essential: partner drug resistance is increasingly common, and, thus, a range of options are needed. Looking to the future, there is still scope for improvement. Current treatments are taken over three days, and compliance with the full course of treatment is poor. A malaria medication allowing a single-dose cure, and, therefore, directly observed therapy, would be ideal. In addition, the relative importance of *P*. *vivax* infections is rising: *P*. *vivax* develops dormant forms or hypnozoites, which result in multiple malaria episodes from a single infection. The ideal medicine for treatment would therefore have activity against the asexual and sexual blood stages of the parasite as well as against the hypnozoites where present *(P*. *vivax* and *P*. *ovale*). In high-transmission areas, such a medicine could provide post-treatment prophylaxis, providing a period of protection for a child after treatment. Although an ambitious target, a single-encounter, radical cure would be of great benefit in malaria elimination, circumventing many issues on cost, logistical management, and adherence issues [10,11]. One critical success factor is that any new compound being developed must be active against preexisting resistant strains. There is also a need for cost-effective malaria chemoprophylaxis for vulnerable groups such as small infants and pregnant women. Increasingly, as the eradication agenda proceeds, there will be a need to protect people from malaria-free districts travelling to areas of high endemicity: for example, in Zambia, travelers from the south of the country are hardly exposed to infection, have little immune protection, and, thus, are at a high risk of infection when travelling to the highly endemic north.

### New Blood Schizonticides in Phase II

Currently, there are four new medicines that have reached clinical Phase II, where they have been shown to be active in curing malaria: OZ439, a third-generation endoperoxide; KAE609, an inhibitor of the parasite sodium channel PfATP4; KAF156, whose mechanism of resistance includes the previously unannotated cyclic amine resistance locus; and DSM265, an inhibitor of the parasite dihydroxyorotate dehydrogenase (DHODH). All four products, which have been discovered within the last decade, are fully active against primary clinical isolates, including the recently characterized artemisinin-resistant strains [12]. They are all relatively fast-acting compounds, but, in contrast to artemisinin, all compounds have long half-lives and show potential to give coverage for over a week from a single dose. The challenge moving forward is to identify the best possible combinations. One option is to go with existing partner drugs such as piperaquine, which is well understood clinically, but for which resistant field isolates exist. The second option is to partner with newer members of the old families: ferroquine or pyronaridine, which have lower risk of resistance. The final option is to pair two new drugs. All three approaches are being considered in the current development pipeline: OZ439 is being tested in a Phase IIb combination trial with piperaquine and is about to be tested in another with ferroquine, both in partnership between MMV and Sanofi. Early combination studies with OZ439 and DSM265 are being carried out in controlled human infection models. KAE609 combination studies are planned for 2016 by MMV and Novartis, although the partner drug strategy has not been finalized.

### Transmission Blocking: Low-Dose Primaquine and Beyond

Artemisinin combination therapies are not active against the late-stage (Stage V) gametocytes, and patients can still carry infectious gametocytes even though they are cured of the asexual forms of the parasite. Primaquine has been shown to be active against these gametocytes with a single low dose of 0.25 mg/kg. In October 2012, WHO released an updated policy recommendation, endorsing this regimen in addition to ACTs [13,14]. In contrast, a 2012 Cochrane report concluded that there was not sufficient evidence to determine whether primaquine reduces gametocyte prevalence and density enough to have an effect on transmission in communities where many infected individuals are asymptomatic [8]. Recent reviews of primaquine in G6PD-deficient patients suggest that the single low dose of primaquine is unlikely to cause hemolysis [15], although safety concerns were sufficient that the more effective, higher dose of 0.75 mg/kg was not recommended. Several trials, expected to be reported in 2016, are underway to provide additional evidence that adding a single low-dose treatment of primaquine to ACTs can help reduce transmission of *P*. *falciparum* malaria, as outlined in the Primaquine Roadmap developed by the Malaria Elimination Initiative of the University of California, San Francisco [16]. The newer medicines in the development pipeline have activity in vitro in standard membrane feeding assays (SMFA) at concentrations similar to those achieved clinically. Studies in controlled human malaria infection (CHMI) models are ongoing to confirm whether this activity is seen clinically. If so, the newer generation of drugs could be seen to be primaquine-sparing, to prolong its global utility as an effective drug, or, at best, prevent transmission without any need for primaquine.

### Tafenoquine

The relapses caused by activation of hypnozoites of *P*. *vivax* can be prevented by a 14-day course of primaquine. This obviously presents issues with compliance in asymptomatic patients. In addition, this regimen carries with it a risk of hemolysis in G6PD-deficient patients. Tafenoquine, a new medicine based on the same 8-aminoquinoline scaffold, originally discovered by the Walter Reed Army Institute of Research (WRAIR), has recently shown great promise in Phase II trials and is currently being tested in pivotal Phase III trials by GlaxoSmithKline in partnership with MMV [17]. Tafenoquine use would still require a G6PD diagnostic test. The search for new molecules that can be used to kill hypnozoites without the need for such a test is being intensified, although so far no candidates have been identified.

### Seasonal Malaria Chemoprevention (SMC)

SMC involves administration of treatment on a monthly basis to coincide with the annual peak in malaria transmission. This intervention is highly effective in reducing the incidence of clinical malaria and anemia in young children, and, in 2012, WHO recommended implementation of SMC for children under the age of five in areas of the Sahel subregion of Africa with highly seasonal transmission. This recommendation is now being implemented increasingly in countries of the Sahel. Although less extensively researched, and not yet recommended by WHO, evidence suggests that SMC is as effective in older children [18].

Table 1 contains a description of the potential global drug pipeline in July 2015. Forty-seven medicines are at varying stages in development, with those in translational and developmental phases given in Table 1 [11, 19, 20].

**Table 1:** Global drug pipeline.

Preclinical	Phase I	Phase II	Phase III
Oxaboroles	Sevuparin	KAE609	Trimethoprim-sulfamethoxazole
P218	ACT451840	OZ439 + piperaquine	Tafenoquine
PA21A092	MMV390048	Fosmidomycin + piperaquine	
NPC1161B	CDRI 97/78	OZ439 + ferroquine	
SJ557733		Methylene blue	
DDD107498		AQ13	
GSK030		DSM265	
AZ412		KAF156	
		Artemisone	

### Vaccines

Vaccines are generally classified into three approaches: pre-erythrocytic vaccines aim to prevent blood-stage infection; blood-stage vaccines aim to clear parasitaemia and prevent clinical disease; and transmission-blocking vaccines aim to prevent infection of mosquitoes and interrupt malaria transmission in populations. There is a growing appreciation that vaccines combining multiple targets and stages will be required for achieving and sustaining elimination.

The development of effective malaria vaccines has been a major goal of the malaria research community for many decades. In 2006, the global Malaria Vaccine Technology Roadmap established the goal of developing an 80%-effective vaccine against *P*. *falciparum* malaria by 2025 that would provide protection for longer than four years, with an interim landmark of a 50%-effective vaccine of one-year duration by 2015 [21]. The new Technology Roadmap updated in 2012 outlines that by 2030, vaccines should be developed that provide at least 75% protective efficacy against clinical malaria, reduce transmission of the parasite, and can be deployed in mass campaigns [22].

Because of the complex life cycle of the *Plasmodium* parasite, host immune response efforts have been focused most recently on the pre-erythrocytic stage of infection to protect against the early stage of malaria infection and thereby block disease progression to red blood cells and clinical malaria [23]. Still, the potential of transmission-blocking vaccines and the need for *P*. *vivax* vaccines in malaria elimination is increasingly being recognized.

RTS,S, a pre-erythrocytic vaccine for the prevention of clinical *P*. *falciparum* malaria in children, is the first vaccine to successfully complete a Phase III clinical trial. The vaccine has recently been approved by the European Medicines Agency, and WHO was imminently expected to make the first malaria vaccine policy recommendations. The trials, conducted in African children, demonstrated a vaccine efficacy for clinical malaria of 50% in children aged 5–17 months old but only 30% in infants, the target population [24]. While RTS,S demonstrates that a malaria vaccine is possible, an ideal candidate to support global eradication efforts would need to have a higher efficacy [25].

A highly effective transmission-blocking vaccine with the ability to interrupt transmission of the *Plasmodium* parasite from infected humans to naïve mosquitoes would be a key tool for malaria elimination [26], but such a vaccine would require an extensive research-and-development agenda. This includes consensus among experts regarding the clinical and entomological metrics required to assess whether a change in transmission has occurred, as well as the tools needed to effectively measure the end point of reduced transmission of parasites to mosquitoes. A standalone transmission-blocking vaccine offers protection at the community level, but does not offer the vaccinated individual protection against becoming infected by *Plasmodium*. Therefore, the ideal vaccine would be a combination of vaccines that protects individuals against disease and/or *Plasmodium* infection and stops transmission of multiple species (and strains), especially the most widespread, *P*. *falciparum* and *P*. *vivax*. Such combination vaccines are yet to be developed and will not be commercially available in the next decade (Table 2).

**Table 2:** Current malaria vaccine clinical trial pipeline.

Vaccine type	Phase Ia	Phase Ib	Phase IIa	Phase IIb	Phase III
	ChAd63/MVA ME-TRAP + Matrix M	Ad35.CS	Ad35.CS/RTS,S-AS01	ChAd63/MA ME-TRAP	RTS,S-AS01
Pre-erythrocytic	Polyepitope DNA EP 1300	PfSPZ	AD35.CS/Ad26.CS		
	PfCelTOS FMP012		ChAd63/MCA		
	CSVAC		M3V.Ad.PfCA		
			M3v.d/Ad.PfCA		
	ChAd63.AMA/MVA.AMA1 + AI/CPG7909	EBA 175.R2	ChAd63/MVA MSP 1	GMZ2	
Blood Stage	SR11.1	SE36	ChAd63.AMA1/MVA.AMA1	MSP3	
	ChAd63/MVA PvDBP *		FMP2.1-AS01B		
Transmission-blocking	Pfs25-EPA				
	Pfs25-VLP				

* Targets *P*. *vivax*

Of the 25 malaria vaccine projects in global development, four are in Phase IIb or III trials [26,27]. All but one current vaccine candidate target *P*. *falciparum* alone, which will likely not be sufficient given the increasing proportion of patients with *P*. *vivax* or mixed infections. Then, there are three other species to be considered: *P*. *malariae*, *P*. *ovale*, and *P*. *knowlesi*, the latter being a zoonotic species.

## Public Health Insecticides

Pyrethroids developed in the 1970s and 1980s are predominantly used for IRS and LLINs. As these interventions have been scaled up, particularly in Africa, over the last decade, pyrethroid resistance has gone from rare frequency and low-level in *Anopheles gambiae* to Africa-wide and high-level, and, in some instances, greater than 1,000-fold levels of resistance compared to susceptible insects [28]. The IVCC was established in 2005 in response to the problem of insecticide resistance and the pressing need for an alternative long-lasting insecticide. The portfolio of products since established is given in Table 3. Syngenta, in collaboration with the IVCC, has reformulated an organophosphate insecticide, Actellic 300CS, to double the longevity of the effective treatment period [29,30]. This product is rapidly replacing pyrethroids for IRS, but, as the product is inherently more expensive, it has cost implications. In addition, replacing one monotherapy, which is the exclusive use of one chemical class, with another will inevitably lead to the failure of this insecticide without careful management [31].

**Table 3:** Current vector control pipeline.

Application	Research	Field Testing	WHO Pesticide Evaluation Scheme (WHOPES) Testing
Indoor Residual Spray			Chlofenapyr
Long-Lasting Indoor Residual Spray		Alphacypermethrin SB	
Long-Lasting Insecticide-Treated Bednet	Repurposed active ingredients from crop protection	Akanet LN	Chlorfenapyr/pyrethroid
	New active ingredients	Miranet LN	Pyriproxyfen/pyrethroid
	Repurposed crop protection compounds	Olyset Duo LN	DawaPlus 2.0 LN
		Panda Net 2.0 LN	Olyset Plus LN*
		Veeralin LN	PermaNet 3.0 LN*
		Yahe LN	
ITN Treatment Kit			ICON MAXX*
Larvacide		VectoMax GR	

* WHOPES Testing and Evaluation Phase III

The urgent requirement is to bring new chemistry to the public health insecticide market. Three new insecticides will be developed from a portfolio of at least nine novel chemistries currently under evaluation [32]. The Innovation to Impact initiative brings together multiple stakeholders to identify mechanisms to effectively shorten the development and registration times for these new products so they reach the market sooner.

It may be possible to reduce selection for resistance with effective integrated vector control. While this strategy is widely promoted, its scale-up potential is limited. Both IRS and LLINs target mosquitoes that bite and rest indoors, but these mainstream interventions do not affect residual outdoor transmission, which is significant in some areas [32,33]. The largest gap in the vector control portfolio is an intervention that can be used at scale to target outdoor resting and outdoor biting mosquito populations. While a number of possible products are being evaluated for the outdoor market, there is no single obviously scalable intervention that can be recommended today.

### Other Potential Vector Control Methods

A variety of other noninsecticidal intervention methods are under development. These include novel genetic approaches and inheritable bacterial endosymbionts. Insects in which the nuclear genome of maternally inherited endosymbionts, such as Wolbachia, have been altered, are released into the target field populations of *Anopheles*. These approaches aim to reduce transmission by reducing the population size, lifespan, or ability to transmit the malaria parasite. To date, *Aedes* mosquitoes have proven more tractable to these approaches, with large-scale field trials underway to test the feasibility of Wolbachia or tetracycline-sensitive lethal positive feedback loops. In *Anopheles*, progress is being made with homing endonuclease genes (HEGs) [34]. However, mainstream adoption of these tools and technologies is still a mid-term rather than a short-term goal.

## Surveillance and Response

In countries that have reached the elimination stage, defined by WHO as those with less than one diagnosed case of malaria per 1,000 people at risk per year, strong surveillance systems are critical to prevent outbreaks. As malaria transmission declines, cases become concentrated in at-risk populations (“hot pops”) or regions (“hot spots”). Epidemiological surveillance is needed to detect “hot pops” and “hot spots,” which can then be targeted for enhanced malaria control. Surveillance in the elimination context strives to identify and respond promptly to every malaria case, ideally within 24 hours [35]. Several key features characterize an ideal surveillance system for malaria elimination.

Reporting and case investigation must be rapid and complete to identify symptomatic and asymptomatic cases and prevent additional malaria infections. This can be achieved using either active or passive approaches. Passive surveillance may be adequate in areas with historically low transmission and good access to health facilities. Active case detection may be needed for hard-to-reach populations who do not attend health facilities, as well as asymptomatic cases that continue to transmit the parasite in the absence of symptoms.

The most widely adopted surveillance and response approach is reactive case detection (RACD), whereby household members, neighbors, and other contacts of passively detected cases are screened for infection and treated with antimalarials [36–38]. China has taken this a step further and implemented a successful “1-3-7” policy, which defines targets of reporting individual cases within one day, conducting case confirmation and investigation within three days, and rolling out appropriate public health response to prevent further transmission within seven days [39].

Surveillance in an elimination context demands integration of related data, such as census or health survey information, central data storage and management, automated and expert data analysis, and customized outputs and feedback that lead to timely and targeted interventions [4–6]. Robust surveillance systems that integrate and synthesize data across multiple sources and use mobile technology will make actionable data available to decision makers at the local, district, and national levels in near real time. Ideal surveillance systems leverage tools like Geographic Information Systems (GIS), spatial modeling, and SDSS to map and predict malaria transmission risk and guide effective, targeted responses at a local level. In elimination programs, and in preventing the reintroduction of malaria to eliminated areas, mapping the risk of receptivity and vulnerability is essential to target surveillance operations. SDSS, which uses automated analysis of geospatial data related to malaria transmission, holds promise for helping countries in pre-elimination and elimination stages use malaria case data to produce accurate risk maps at finer scales, map intervention coverage, and guide control programs to improve effectiveness and efficiency [7].

Malaria in high-risk populations constitutes one of the most intractable challenges to control efforts. An example is in the Greater Mekong subregion, where massive human migration occurs across porous borders, sustaining and spreading multidrug-resistant *P*. *falciparum* malaria. Often, these groups reside in rural areas with poor access to treatment, are migrant workers, or are economically or politically marginalized. Patterns of human mobility help give country managers critical information about the risk of reintroducing the parasite into areas with little or no malaria.

Surveillance itself is an elimination intervention that reduces transmission if detection of focal outbreaks and treatment of infections from the infectious reservoir can occur rapidly. Taken together, the information provided by a robust surveillance and response system allows resources to be deployed in a timely, targeted, and efficient manner. Operational research to identify the effectiveness of various surveillance strategies is currently underway.

### Mass Drug Administration

Mass screening and treatment using RDTs of populations at high risk of malaria has been shown to be ineffective at reducing malaria transmission, most likely because of the large proportion of infections that are left undetected and untreated. Parasite densities are usually low in asymptomatic individuals, and RDTs as well as PCRs from filter paper blood spots lack sufficient sensitivity to detect low densities.

Recently, there has been renewed interest in the empiric administration of a therapeutic antimalarial regimen to an entire population at the same time, otherwise known as mass drug administration (MDA). This presumptive antimalarial treatment of targeted populations is also called targeted malaria elimination (TME) and targeted malaria treatment (TMT), or targeted parasite elimination (TPE). The potential impact of MDA on malaria is large, reducing the chances of malaria mortality and morbidity through its direct therapeutic or prophylactic effect on individuals who receive a treatment dose of antimalarials. MDA can reduce transmission rates by reducing parasitaemia prevalence and interrupting various stages of the parasite life cycle. Some antimalarial drugs could inhibit the sporogonic cycle in the mosquito, reducing its vectorial capacity. If every member of a given population is treated by antimalarial MDA, an immediate reduction in asexual parasite prevalence in the population would result.

A 2013 Cochrane systematic review concluded that MDA of antimalarials appears to substantially reduce the initial risk of malaria parasitaemia [39], but there remain knowledge gaps, especially on optimal target population size, methods to improve coverage, and primaquine safety [40,41].

Targeted MDA will likely be used to block transmission in foci and accelerate the impact of vector control and access to diagnosis and treatment in malaria elimination programs. One key issue here is that medicines such as ACTs and primaquine have been registered based on a clinically demonstrated risk:benefit ratio in symptomatic patients. The evidence base for an appropriate risk:benefit ratio in asymptomatic or noninfected subjects will need continual attention.

The regimen used for MDA should differ from the first-line treatment in the same area, so that treatment and prophylactic treatment do not add to the selection pressure for resistance on a single drug. Current trials use a full course of dihydroartemisinin/piperaquine (DHA-P), and a single low dose of primaquine is added to the drug combination to abort gametocytaemia. Alternative antimalarials, such as artemether combined with lumefantrine, can be considered, but this partner drug has a shorter half-life. A minimum of three “rounds” of drug administration is needed to ensure an impact on transmission. Preliminary data show that DHA-P is effective in curing asymptomatic parasite carriers, as well as in areas with established artemisinin resistance. With no appropriate alternative antimalarials currently available, there is no real substitute for ACTs for use in TMT. The potential hazard of increasing drug pressure with ACTs by using a mass drug treatment approach on an already resistant parasite population is well recognized.

Complicating deployment, it is not known whether many medicines considered for MDA are safe during first-trimester pregnancy. This presents additional problems if the medicines are deployed in Africa, where pregnancies are not usually reported in the first trimester.

## Getting Ahead of the Resistance Curve

Vigilance for the emergence and spread of drug-resistant parasites and declining treatment efficacy is crucial. While resistance is a major problem, the current response has been reactive rather than proactive. The resistance phenotypes have to be selected before establishing the underlying mechanism of the resistance, followed by simplified methodologies to track resistance as it moves through parasites in insect vector populations and to establish the resistance’s impact on interventions. The advent of modern high-throughput genome sequencing and big data analytics has opened up the potential for a more proactive approach. Genome sequencing allows signatures of selection to be identified as soon as a new intervention is used. The ability to predetermine the genes under selection before an intervention is deployed at scale will allow the design of effective resistance-management strategies to maximize the efficacy of both medicines and insecticides.

## Conclusions

Collectively, the pipeline of new malaria prevention and treatment tools has never been healthier. However, the scale at which current treatments are being deployed has facilitated the widespread emergence of resistance, creating an urgent need for better field diagnostic tools and new methods for quickly, cost-effectively, and accurately detecting and preventing importation of malaria parasites in low-endemic settings. To ensure that these tools are able to contribute effectively to rapid reductions in malaria transmission, appropriate scale-up and roll-out strategies must be developed. Better approaches to surveillance will both identify key sources and pathways of malaria transmission and focus diagnosis, treatment, and prevention resources where they can be most effective in accelerating parasite elimination and saving lives. New strategies will also help to identify the most efficient ways to deploy interventions. If these interventions are properly implemented in an integrated and complementary manner, we will be able to sustain and build upon the impressive reductions that have been achieved to date, significantly enhancing the prospects for malaria elimination.

## Abbreviations

ACTs: artemisinin-based combination therapies
AIM: *Action and Investment to defeat Malaria*
CHMI: Controlled Human Malaria Infection
DHA-P: dihydroartemisinin/piperaquine
DHODH: dihydroxyorotate dehydrogenase
DNDi: Drugs for Neglected Diseases Initiatives
EVI: European Vaccines Initiative
FIND: Foundation for Innovative New Diagnostics
GIS: Geographic Information Systems
GTS: *Global Technical Strategy for Malaria*
HEGs: homing endonuclease genes
IDT: Infection Detection Test
IPTp: intermittent preventive treatment for pregnant women
IRS: indoor residual spraying
IVCC: Innovative Vector Control Consortium
LAMP: Loop-Mediated Isothermal Amplification
LLINs: long-lasting insecticide-treated bed nets
LMICs: low- and middle-income countries
MDA: mass drug administration
MMV: Medicines for Malaria Venture
MVI: Malaria Vaccine Initiative
NITD: Novartis Institute for Tropical Diseases
PATH: Program for Appropriate Technology in Health
PCR: Polymerase Chain Reaction
PDPs: Product Development Partnerships
RACD: reactive case detection
RBM: Roll Back Malaria
RDTs: Rapid Diagnostic Tests
SDSS: spatial decision support systems
SMC: Seasonal Malaria Chemoprevention
SMFA: Standard Membrane Feeding Assays
TME: targeted malaria elimination
TMT: targeted malaria treatment
TPE: targeted parasite elimination
WHOPES: WHO Pesticide Evaluation Scheme
WRAIR: Walter Reed Army Institute of Research
